# Peripheral Nerve Sheath Tumors Resembling Human Atypical Neurofibroma in Goldfish (*Carassius auratus*, Linnaeus, 1758)

**DOI:** 10.3390/ani11092621

**Published:** 2021-09-07

**Authors:** Federico Armando, Claudio Pigoli, Matteo Gambini, Andrea Ghidelli, Gabriele Ghisleni, Attilio Corradi, Benedetta Passeri, Mario Caniatti, Valeria Grieco, Wolfgang Baumgärtner, Christina Puff

**Affiliations:** 1 Department of Pathology, University of Veterinary Medicine Hannover, Bünteweg 17, 30559 Hannover, Germany; federico.armando@tiho-hannover.de (F.A.); gambini.matteo@unimi.it (M.G.); christina.puff@tiho-hannover.de (C.P.); 2 Pathology Unit, Department of Veterinary Medicine, University of Parma, Strada del Taglio 10, 43126 Parma, Italy; attilio.corradi@unipr.it (A.C.); benedetta.passeri@unipr.it (B.P.); 3 Dipartimento di Medicina Veterinaria (DIMEVET), Università degli Studi di Milano, Via dell’Università 6, 26900 Lodi, Italy; claudio.pigoli@unimi.it (C.P.); ghisleni@ticino.com (G.G.); mario.caniatti@unimi.it (M.C.); valeria.grieco@unimi.it (V.G.); 4 Laboratorio di Istologia, Sede Territoriale di Milano, Istituto Zooprofilattico Sperimentale della Lombardia e dell’Emilia-Romagna (IZSLER), 20133 Milano, Italy; 5 Department of Veterinary Medicine, University of Parma, Strada del Taglio 10, 43126 Parma, Italy; drghidellivet@gmail.com; 6 Biessea Laboratorio Analisi Veterinarie, Via Amedeo D’Aosta 7, 20129 Milano, Italy

**Keywords:** Azan trichrome stain, CNPase, reticulin stain, S100

## Abstract

**Simple Summary:**

In animals, especially in fish, dermal neoplasms are a common finding. A distinction between peripheral nerve sheath tumors (PNSTs) and other spindle cell tumors (SCTs) is not always possible when relying exclusively on routine cytological and histopathological findings. The current study aims to determine a minimal subset of stains required to correctly identify PNSTs in goldfish and describes, in detail, six dermal nodules that resemble atypical neurofibroma in humans. Interestingly, muscular and fibroblastic tumors were excluded using Azan trichrome staining, while Alcian blue and Gomori’s reticulin stains revealed the presence of intratumoral areas of mucins and basement membrane fragments, respectively. In addition, PAS and PAS with diastase pretreatment confirmed the latter finding and revealed intra- and extracellular glycogen granules. Immunohistochemistry displayed reactivity for S100 protein, CNPase, and phosphorylated and non-phosphorylated neurofilament-positive axons. Altogether, these findings suggested that Azan trichrome staining, Gomori’s reticulin staining, and immunohistochemistry for S100 protein and CNPase represent a useful set of stains to identify and characterize PNSTs in goldfish.

**Abstract:**

Skin spindle cell tumors (SSTs) frequently occur in fishes, with peripheral nerve sheath tumors (PNSTs) being the most commonly reported neoplasms in goldfish. However, distinguishing PNSTs from other SCTs is not always possible when relying exclusively on routine cytological and histopathological findings. Therefore, the aim of this study is to characterize six skin nodules, resembling atypical neurofibromas in humans, found in six cohabiting goldfish (*Carassius auratus*), and to determine a minimal subset of special stains required to correctly identify PNSTs in this species. Routine cytology and histopathology were indicative of an SCT with nuclear atypia in all cases, with randomly distributed areas of hypercellularity and loss of neurofibroma architecture. Muscular and fibroblastic tumors were excluded using Azan trichrome staining. Alcian blue and Gomori’s reticulin stains revealed the presence of intratumoral areas of glycosaminoglycans or mucins and basement membrane fragments, respectively. PAS and PAS–diastase stains confirmed the latter finding and revealed intra- and extracellular glycogen granules. Immunohistochemistry displayed multifocal, randomly distributed aggregates of neoplastic cells positive for S100 protein and CNPase, intermingled with phosphorylated and non-phosphorylated neurofilament-positive axons. Collectively, these findings are consistent with a PNST resembling atypical neurofibroma in humans, an entity not previously reported in goldfish, and suggest that Azan trichrome staining, reticulin staining, and immunohistochemistry for S100 protein and CNPase represent a useful set of special stains to identify and characterize PNSTs in this species.

## 1. Introduction

Neoplastic diseases are not exclusive to domesticated mammals [1] but are frequently reported to affect small mammal pets [2], reptiles [3], and fishes [3,4,5,6,7]. 

Fish breeding is a constantly expanding business, carried out with seafood production, research, exhibition, and ornamental purposes. Aquaculture is, nowadays, an industry facing yearly growth all around the world [8]. Fish are also commonly used as laboratory animals, representing an important animal model for toxicological research [9] as well as for carcinogenesis and cancer development studies [10,11,12,13]. Finally, an economically promising niche is represented by exhibition fish breeding, with valuable animals reaching remarkable prices among hobbyists [14]. 

Spontaneous neoplasms have been reported in fishes bred in aquaculture, in laboratory facilities, and in public and private aquaria, as well as in piscine wildlife [3,4,5,7,15,16,17,18]. The increasing number of reports regarding tumors in fishes is based on the role of these animals as sentinels for oncogenic environmental pollution, their growing impact as laboratory models, and the progressive extension of the life span of exhibition subjects [3,5,7]. Among fishes, tumors evolving from skin and subcutaneous tissue are commonly reported [4,6,7,16,19]. This is probably correlated with the fact that water could represent an efficient vehicle for chemical agents (i.e., food toxicants, water pollutants), viruses, mycobacteria, and parasites [3,5,6,7]. Indeed, all these factors have been correlated with the occurrence of neoplasms in fishes, besides chronic trauma and/or inflammation and UV radiation [3,4,5,7,10,11,20,21,22,23,24]. Moreover, most fish are ectotherms [25] and, therefore, are highly sensitive to temperature variations, which can act as predisposing factors for tumor development [4,20,24]. Neoplasms can represent a life-threatening condition in these animals due to both the infiltrative behavior of the tumor and the occurrence of secondary infections following tumor ulceration [4,5,7,26]. Piscine neoplasms share many features with their mammalian counterparts, including a different biological behavior depending on the degree of differentiation [3,27,28]. However, different from what has been reported in mammals [1], metastasis occurs only rarely in fishes [3,4,5,7,11]. Gross differential diagnoses for piscine skin nodules include both epithelial and mesenchymal neoplasms as well as traumatic hematomas, foci of hyperplasia and/or hyperpigmentation, xanthomas, granulation tissue, granulomas, lymphocystis disease, and parasitic lesions [3,4,5,6,29].

Koi and goldfish, both members of the family Cyprinidae of teleosts, have always been among the most common exhibition and ornamental fishes [14]. Therefore, a large number of different spindle cell tumors have been reported in these animals, including peripheral nerve sheath tumors (PNSTs) [7,19,29,30,31,32], chromatophoromas [26,33], fibroma/fibrosarcoma [34,35,36,37], leiomyoma [38,39], myxoma [37], and perivascular wall tumors (PWTs) [40,41]. PNSTs can be further divided into benign PNST (BPNST) and malignant PNST (MPNST), with the former further categorized as Schwann cell tumor, neurofibroma, perineurioma [1,30,42,43,44,45,46], or myxoma of the dermal nerve sheaths [47,48]. Specifically, several PNST variants (i.e., classical schwannoma, neurofibroma, pigmented variants of both aforementioned and malignant schwannomas) have been reported in goldfish [19,30,31,32]. However, the differentiation of the various BPNST subtypes is not always straightforward [30,31,32,44,49,50,51]. In contrast to domestic mammals [1], immunohistochemical panels to characterize skin tumors, as well as their biological behavior and clinical outcome, are not widely and systematically described for fishes. Nonetheless, the use of histochemical and immunohistochemical stains to support morphological evaluation, despite not always being successful, has gained a pivotal role in fish pathology [26,27,30,39,52,53], besides ultrastructural investigations, with the aim of supporting the final diagnosis [4,5,6,33,54].

In this context, the aim of the current study is to characterize skin nodules surgically excised from a group of six goldfish (*Carassius auratus*) cohabiting in the same pond. The nodules, which were cytologically and histologically consistent with spindle cell tumors, resembling atypical neurofibroma in humans, further underwent a panel of histochemical and immunohistochemical stains to identify a set of special stains and/or markers useful to confirm the diagnosis of PNST, which represents one of the most commonly reported neoplasms in goldfish [7,19,29,30,31,32].

## 2. Materials and Methods

### 2.1. Clinical Findings, Anesthesia, Surgery, and Sample Collection 

One adult goldfish was referred to a veterinary physician (A.G.) for an exophytic, approximately 0.5 cm in diameter, whitish nodule arising from the lateral trunk. The goldfish belonged to a group of 6 adult animals of the Ryukin strain, ranging from 12 to 15 cm in length, of unknown sex, obtained from the same breeding batch (i.e., a group of >2 males and >2 females bred in the same tank). The fish were the only animals living on the property of the owner and were managed according to high-quality breeding standards [14]. Specifically, the animals were housed in a 250 L external artificial pond placed in an adequately illuminated spot, despite not being directly exposed to the sunlight. The water, which was obtained from a tap and had features compatible with human consumption (according to the Italian law D.lgs 31/01), was cleaned with a mechanical filter, the sponges of which were washed every three days in conjunction with a pond water change. No chemical treatments (such as acidity regulators or anti-parasite medications) were added to the water. At clinical inspection, the other five goldfish also revealed the presence of skin nodules, although markedly smaller than the one from the first animal. All six goldfish were in good general health condition and did not show any other external lesions, including the presence of ectoparasites. 

Three weeks after the first clinical inspection, all the nodules variably increased in size. Therefore, the nodules were surgically excised for further diagnostics after obtaining the written consent of the owner. 

Anesthesia was induced via immersion of the animals in an aliquot of water from the pond, containing 10 mg/L propofol (Propofol-Lipuro 20 mg/mL; B. Braun Melsungen AG, Melsungen, Germany) and oxygenated with an airstone [55]. Maintenance of anesthesia was performed with an artificial non-recirculating ventilation system directly applied to the gills, delivering water from the fish’s pond added with 7.5 mg/L propofol and adequate oxygen [25]. Analgesia was locally provided with multiple injections of 2% lidocaine (Fisiopharma, Palomonte, Salerno, Italy) diluted 1:100 in NaCl 0.9%, administered encircling and under each nodule [5]. 

Prior to surgical excision, fine needle aspirates of all the six nodules were performed, and the obtained smears were air-dried. The nodules were then excised at the base [4,5], measured, and fixed in 10% neutral buffered formalin for histopathological examination. Stomahesive^®^ Protective Powder (ConvaTec Group PLC, Reading, Berkshire, UK) and diluted iodopovidone (Betadine; Purdue Pharma LP, Stamford, CT, USA) were finally administered over the wounds without suturing [4]. No prophylactic antibiotic treatment was administered [4]. 

Recovery was obtained via immersion of each fish in anesthetic-free, adequately oxygenated water from the fish pond. Full recovery was delayed in two animals, which showed poor buoyancy in the next 4 days, followed by a return to normal condition without any treatment. 

At 180 days after surgery, 2 animals were healthy, while 4 animals died 150 days after surgery due to unknown causes. Necropsy of the dead animals was not performed, but a biopsy of an umbilicate scar that had risen at the surgical site of one of those fish (Case 6) was submitted for histopathological evaluation. 

### 2.2. Cytological and Histopathological Sample Processing and Evaluation

Cytological samples were stained with May-Grünwald-Giemsa according to the manufacturer’s instructions (Bio-Optica Milano SPA, Milano, Italy) and evaluated with bright field microscopy. 

Following 24 h of fixation in 10% neutral buffered formalin, the tissue samples were routinely processed for histopathology and embedded in paraffin wax (FFPE). Vertical sections of the neoplasms, including the overlying skin of 3 µm, were stained with hematoxylin and eosin (HE) and evaluated with bright field microscopy (Olympus IX-70, Olympus Optical Co. GmbH, Hamburg, Germany).

### 2.3. Histochemical and Immunohistochemical Stains 

Additional sections for each nodule were stained with the following histochemical stains: Azan trichrome, Alcian blue (pH 2.5), Gomori’s reticulin, Bielschowsky stain, and Ziehl-Neelsen stain, as well as with periodic acid-Schiff stain (PAS) with and without diastase pretreatment. For each special stain, several tissues (i.e., eye, brain, spinal cord, peripheral ganglion, peripheral nerve, skeletal muscle, liver, pancreas, digestive tract, skin) obtained from a goldfish unrelated to the study and without significant histopathological findings, as well as appropriate canine tissues, were included as positive controls. Only histochemical stains exhibiting cross-reaction with the expected target tissue(s) from the control goldfish were included in the study.

The same set of unaltered goldfish tissues employed as positive controls for histochemical stains was also used for the preliminary evaluation of immunohistochemical (IHC) cross-reactivity with piscine tissues of several primary antibodies, adding to each reaction an appropriate canine tissue as positive control (Table S1). Tested primary antibodies were directed against α-smooth muscle actin (α-SMA), c-Kit/CD117, calretinin, chromogranin-A, 2′,3′-cyclic-nucleotide phosphodiesterase (CNPase), desmin, glial fibrillary acidic protein (GFAP), Ki-67, EGR2/Krox20, melan-A, non-phosphorylated neurofilament (n-NF), neuron-specific enolase (NSE), phosphorylated neurofilament (p-NF), periaxin (PRX), PNL-2, p75NTR/nerve growth factor receptor (NGFR), SOX2, synaptophysin, S100 protein, and vimentin. Host species and clonality of each tested primary antibody, as well as the corresponding antigen retrieval, serum blocking, dilution, secondary antibody, and appropriate piscine and mammalian positive controls, are reported in Table S1. IHC stains were performed as previously described [56], with slight modifications. Briefly, 3 µm thick sections of formalin-fixed and paraffin-embedded tissues underwent inhibition of endogenous peroxidases and, when necessary, were pretreated for antigen retrieval, as described in Table S1. Then, serum blocking followed by overnight incubation with primary and corresponding biotinylated secondary antibodies were performed. The formation of antigen–antibody complexes was visualized by applying the ABC method, with 3,3′-diaminobenzidine-tetrahydrochlorid (DAB) as a chromogen. Finally, counterstaining with Mayer’s hematoxylin was performed. For negative controls, each rabbit polyclonal or mouse monoclonal primary antibody was replaced with rabbit serum or Balb/c ascitic fluid at the corresponding protein concentration, respectively.

IHC staining was performed on 3 µm thick sections of each surgically excised nodule only for those primary antibodies exhibiting a cross-reaction with the expected target tissue(s) from the control goldfish. Additionally, in this phase, appropriate goldfish and mammalian tissues were included as positive controls for each IHC staining. 

Immunohistochemically stained sections of the surgically excised nodules were evaluated in a semi-quantitative manner: − (0% positive cells), + (0–25% positive cells), ++ (26–50% positive cells), +++ (51–75% positive cells), and ++++ (76–100% positive cells). The distribution pattern (e.g., multifocal, diffuse) of positive cells and the intracellular localization (i.e., membranous, cytoplasmic, nuclear) of each marker were also reported.

## 3. Results

### 3.1. Macroscopical and Cytological Evaluation

**Figure 1:**
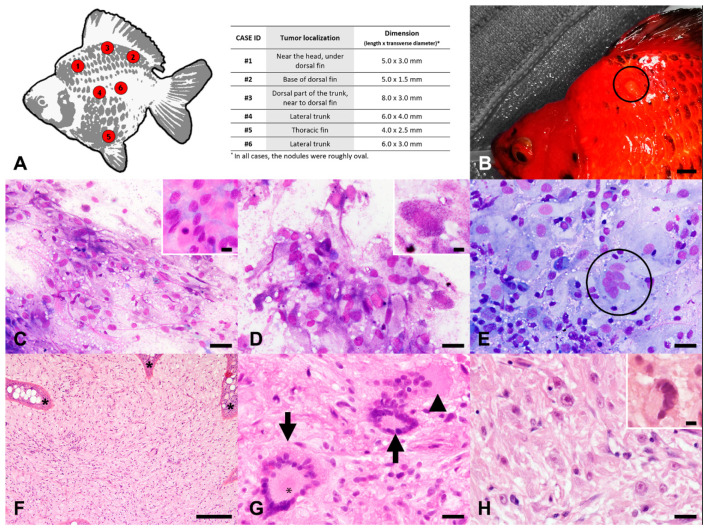
Atypical neurofibroma, skin, goldfish. (**A**) Localization of the nodules (left) and corresponding size (table on the right). (**B**) Case 1: Gross appearance of one of the nodules in situ (encircled). (**C**) Case 1: Fine needle aspiration smear of the nodule with discrete or loosely cohesive, spindle-shaped to polygonal-shaped atypical cells embedded in a finely fibrillar, compact, and pinkish extracellular matrix (insert). May-Grünwald-Giemsa (MGG). (**D**) Case 1: Atypical cells with small, magenta intracytoplasmic granules (insert). MGG. (**E**) Case 6: Presence of occasional multinucleated cells (encircled) within the tumors. MGG. (**F**) Case 3: Intradermal neoplasm composed of cells arranged in streams, loosely arranged interlacing bundles, whorls, or in a storiform pattern with hyperplasia of the overlying epidermis with rete ridge formation (asterisks). Hematoxylin and eosin (HE). (**G**) Case 3: Multifocal aggregates of cells palisading around a central area filled with fine eosinophilic fibrils lined by cell cytoplasms (asterisk), resembling rosettes of the pineocytomatous/neurocytic type (arrows), and a giant multinucleated neoplastic cell (arrowhead). HE. (**H**) Case 2: Spindle-shaped to polygonal-shaped tumor cells displayed moderate to marked anisocytosis, anisokaryosis, and macronucleolosis with prominent magenta nucleoli, with the occasional presence of atypical, large, hyperchromatic nuclei with smudgy chromatin (insert). HE. Scale bars: 0.5 cm (**B**), 100 µm (**F**), 20 µm (**C**–**E**,**G**,**H**), 10 µm ((**C**) insert, (**D**) insert, (**H**), insert).

A single skin nodule was surgically excised from each animal, originating from different localizations (Figure 1A). Macroscopically, the six variably sized lesions were roughly oval, as summarized in Figure 1A. The tumors were non-ulcerated, well-circumscribed, and slightly raised above the skin surface (Figure 1B). On the cutting surface, the masses were yellow to orange, with a friable texture, and well-circumscribed, with no clear evidence of infiltrative growth. 

The detailed results of the cytological evaluation of each case are summarized in Table S2. Cytological samples were moderately to highly cellular, with generally well-preserved cells except for Cases 3 and 4, which were characterized by numerous naked nuclei. All specimens displayed a mild hemodilution and a glassy, lightly bluish background, with scant to numerous small slightly to intensely magenta granules of unknown origin, admixed with a variably abundant meshwork of finely fibrillar, compact, and pinkish extracellular matrix (Figure 1C). 

Cytological samples from Cases 1 and 6 were mixed, with a prevalent population (70%) of discrete or loosely cohesive atypical cells (Figure 1C) admixed with epithelial cells intermingled with fewer club cells, reactive and foamy macrophages, and lymphocytes. In the specimens obtained from Case 1, occasional melanophores associated with scant small gray-bluish granules on the background (consistent with melanophore granules) were also present. Atypical cells were spindle- to polygonal-shaped, up to roundish-shaped (Figure 1D), with a maximum diameter of 55 µm. Cells had variably distinct borders, an intermediate nuclear to cytoplasmic ratio, and a moderately abundant, homogeneous, weakly bluish cytoplasm containing scant to numerous small, slightly to intensely magenta granules (Figure 1D), morphologically consistent with those found in the background. Nuclei were central, oval to round, 28 µm in maximum diameter, with finely stippled to finely reticular chromatin and no evident nucleoli. Anisokaryosis and anisocytosis were moderate. Mitoses were not observed. Variable amounts of binucleated and multinucleated atypical cells (Figure 1E) were also present. 

Cytological specimens from Cases 2, 4, and 5 were characterized by a mixed cellular population, mostly consisting of large clusters (75–85%) of epithelial cells with moderately to intensely blue cytoplasms and intermingled with fewer club cells, admixed with discrete or loosely clustered atypical cells (5–15%), reactive and foamy macrophages, and lymphocytes. In Case 3, a lower proportion of discrete or clustered epithelial cells (30%) was associated with a marked increase of reactive and foamy macrophages (30%) and lymphocytes (20%) compared to the other cases, admixed with viable neutrophils (10%). Discrete or loosely clustered atypical cells (10%) were also present. Atypical cells found in Cases 2, 3, 4, and 5 were morphologically consistent with those found in Cases 1 and 6, with mild anisokaryosis and anisocytosis and no evident mitoses. Occasional binucleated atypical cells were evident only in Case 3.

On the basis of the cytological findings described above, the samples from Cases 1 and 6 were diagnosed as highly suggestive of a spindle cell neoplasm. On the other hand, the amount of atypical cells in cytological samples from the other cases was too low to suspect an underlying neoplastic process. Therefore, the cytological diagnosis was epidermal hyperplasia for Cases 2, 4, and 5, with the addition of lymphohistiocytic inflammation in Case 3. Neither fungal organisms nor bacteria were evident in any of the cases examined. 

### 3.2. Histopathological Evaluation

The detailed results of the histopathological evaluation of each case are summarized in Table S3. Histopathological examination of samples from all cases revealed a predominantly intradermal mass expanding from the subcutis up to the dermo-epidermal junction, effacing 80–90% of each section (Figure 1F). The moderately cellular, ill-demarcated, non-encapsulated, expansile masses extended to the excision margins and were associated with effacement of the distinction between the dermal stratum spongiosum and stratum compactum, with complete loss of dermal scales (Figure 1F). 

Each nodule was composed of cells growing in streams and loosely arranged interlacing bundles (70%) in whorls (15%) or a storiform pattern (15%) (Figure 1F). Occasional areas with increased cellularity and a fascicular pattern were also present. In Case 3, multifocal aggregates of cells palisading around a central area lined by cell cytoplasms and filled with eosinophilic fine fibrils and occasional round, 2–4 µm, hyaline, and eosinophilic granules were additionally evident (interpreted as rosettes resembling the pineocytomatous/neurocytic type; Figure 1G). Neoplastic cells were supported by a moderate amount of a moderately compact, haphazardly arranged fibrillary stroma with scattered small- to medium-sized blood vessels (Figure 1F,H). In Cases 1, 3, 4, and 6, multifocal to locally extensive hypocellular foci, characterized by interstitial depositions of myxoid material, were present within the neoplasm. 

Neoplastic cells were spindle- to polygonal-shaped, up to 40 µm long and 18 µm wide, with variably distinct borders and an intermediate nuclear–cytoplasmic ratio (Figure 1G,H). The cytoplasm was moderately abundant, homogeneous to finely granular, and pale eosinophilic. The central to paracentral, oval to round, and up to 22 µm in maximum diameter nucleus displayed variably evident indentations (more prominent in Sase 2), finely granular to vesicular chromatin, and 1–2 variably prominent, paracentral, round, 1–2 µm-sized, basophilic nucleoli. 

Anisokaryosis and anisocytosis were moderate to marked. Mitotic count ranged from 0 (Cases 1, 4, and 5) to 1 (Cases 2, 3, and 6) per 10 high power fields. Disseminated throughout the neoplasms were single neoplastic cells characterized by an atypical, large, hyperchromatic nucleus with smudgy chromatin (nuclear atypia) or a regular nucleus with marginated chromatin containing an oval, 3–5 µm-sized, prominent magenta nucleolus (macronucleolosis; Figure 1H). In Cases 2 and 3, occasional bi- and trinucleated, round to polygonal, up to 35 µm-sized neoplastic cells were evident, especially in the superficial dermis containing an abundant, finely granular eosinophilic cytoplasm. In Case 3, a few discrete, multinucleated giant neoplastic cells, ranging from 40 to 150 µm in size, with up to 30 haphazardly distributed nuclei displaying moderate anisokaryosis and a finely vacuolated eosinophilic cytoplasm, were also present (Figure 1G). 

Disseminated throughout the neoplasms and occasionally (Case 6) arranged around small-caliber blood vessels were low to moderate numbers of viable neutrophils and heterophils admixed with mature small lymphocytes. In Cases 3 and 6, multifocal, mild intratumoral hemorrhages were present, with few hemosiderophages, which were most prominent around small-caliber vessels. Scattered melanophores, most numerous in Case 2, were also evident throughout the nodules. 

In Cases 3, 4, and 6, the epidermis overlying the nodules was diffusely thickened by marked hyperplasia of the epithelial cells, with moderate to marked hyperplasia of both goblet cells and club cells, characterized by the formation of multifocal extensions projecting into the neoplasm (rete ridges). The epidermis overlying the nodules in the other cases was characterized by locally extensive erosions (Cases 2 and 5) and/or microulcerations (Cases 1 and 5), with the occasional infiltration of low numbers of mature small lymphocytes, viable and degenerated neutrophils and heterophils and fewer macrophages. 

For Case 6, a second biopsy obtained from a scar from the surgical excision of the neoplastic nodule was examined. The full-thickness skin biopsy was characterized by multifocal, large subepidermal areas effaced by neoplastic tissue with morphological features, reminiscent of those of the nodules described above. The overlying epidermis was mildly hyperplastic and characterized by a reduced number of goblet cells and club cells (consistent with wound healing). In the deep dermis were multifocally extensive foci of fibroplasia and fibrosis intermingled with increased amounts of adipose tissue. The underlying skeletal muscles were multifocally characterized by the hyaline degeneration and fragmentation of myofibers, occasionally associated with necrosis of the myocytes and infiltration of moderate numbers of fibroblasts and few foamy macrophages.

On the basis of similar histopathological findings, all six nodules were diagnosed as dermal spindle cell tumors with nuclear atypia and randomly distributed areas of hypercellularity. According to the morphological features, differential diagnoses included peripheral nerve sheath tumor, fibroma/fibrosarcoma, and undifferentiated chromatophoroma. To further characterize the tumors, several histochemical and immunohistochemical stains were performed. 

### 3.3. Histochemical Stains

All the histochemical stains attempted on the FFPE sections of the neoplasms exhibited cross-reaction with the expected target tissue(s) from the control goldfish and were, therefore, included in the study. The results are summarized in Table 1. 

**Figure 2:**
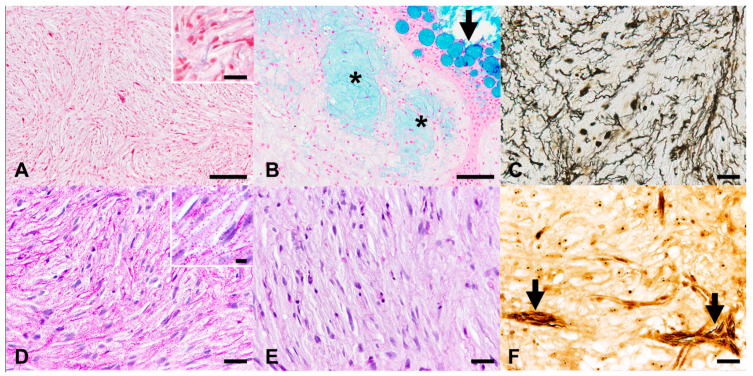
Atypical neurofibroma, skin, goldfish. (**A**) Case 6: Lack of magenta staining of tumor cells, excluding muscular cells as the eventual origin of the neoplasm. Presence of low numbers of collagen fibers (blue) within the tumors (insert). Azan trichrome stain. (**B**) Case 4: Presence of interstitial deposition of glycosaminoglycans or mucins (asterisks) within the tumors. Epidermal goblet cells served as internal positive controls (arrow). Alcian blue stain (pH 2.5). (**C**) Case 6: Scattered to disseminated, single, wrinkling, short fibrils, consistent with basement membrane fragments. Gomori’s reticulin stain. (**D**,**E**) Case 6. (**D**) Presence of numerous PAS-positive granules within neoplastic cells (insert), which disappeared after diastase treatment (**E**), suggestive of glycogen granules. PAS (**D**) and PAS-diastase (**E**) stain. (**F**) Case 2: Presence of silver-impregnated thin fibers in two of the nodules (arrows), suggestive of axonal elements. Bielschowsky stain. Scale bars 100 µm (**A**,**B**), 20 µm ((**A**) insert, (**C**–**F**)), 10 µm ((**D**) insert).

In Cases 5 and 6, Azan trichrome staining revealed the presence of only scant, single, short collagen fibers scattered throughout the tumors (Figure 2A). Furthermore, differently from the control tissues, in which smooth and striated muscular fibers were stained magenta, neoplastic cells did not exhibit this color in all examined cases. 

Alcian blue staining (pH 2.5) showed positively stained intratumoral areas corresponding to the hypocellular foci of myxoid material observed in HE sections (Figure 2B). This observation is consistent with the interpretation of these findings as glycosaminoglycan or mucin depositions. 

Gomori’s reticulin staining revealed, in all cases, the presence of scattered to disseminated, single, wrinkling, short fibrils consistent with basement membrane fragments (Figure 2C). 

Similar to Gomori’s reticulin staining, PAS staining revealed the presence of scattered to disseminated thin lamellar structures, partially or entirely lining individual neoplastic cells, which were interpreted as basement membrane fragments (Figure 2D). Additionally, variable numbers of extracellular and intracellular PAS-positive, diastase-sensitive granules were present (Figure 2D,E), thus being consistent with glycogen. 

Bielschowsky staining revealed occasional silver-impregnated thin fibers in two nodules (Figure 2F), thus suggesting the presence of axonal elements. 

Ziehl-Neelsen staining did not reveal intralesional acid-fast bacteria in any of the cases.

### 3.4. Immunohistochemistry

Primary antibodies directed against α-SMA, chromogranin-A, desmin, Ki-67, melan-A, NSE, PRX, p75NTR/NGFR, synaptophysin, and vimentin did not cross-react with the expected target tissue(s) from the control goldfish. Therefore, these antibodies were excluded from the current study and not tested on tumor FFPE sections. 

**Figure 3:**
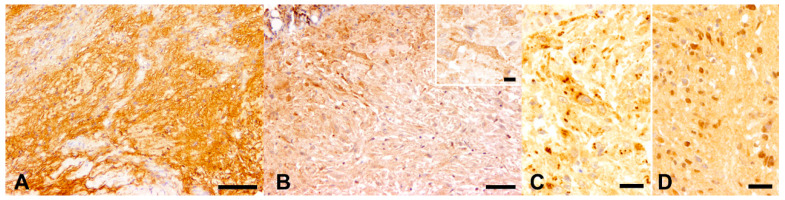
Immunohistochemistry (IHC) of atypical neurofibroma, skin, goldfish. (**A**) Case 1: Patchy distributed neoplastic cells with a cytoplasmic expression of S100 protein. IHC for S100 protein. (**B**) Case 2: Variable numbers of neoplastic cells with a membranous to cytoplasmic expression of CNPase (insert). Positive cells were mostly located in the superficial dermis, progressively reducing in number towards the bottom part of each nodule. IHC for CNPase. (**C**,**D**) Tumor cells displaying variably distributed positivity for non-phosphorylated neurofilaments (**C**, Case 2) and scattered positivity for phosphorylated neurofilaments (**D**, Case 4). IHC for non-phosphorylated (**C**) and phosphorylated (**D**) neurofilaments, focal cytoplasmic immunolabeling. Scale bars: 100 µm (**A**), 50 µm (**B**), 20 µm (**C**,**D**), 10 µm ((**B**) insert).

The results of IHC stains performed on tumor FFPE sections are summarized in Table 2. In each case, IHC for S100 protein revealed a variable number of neoplastic cells exhibiting a strong cytoplasmic expression; they were not homogeneously distributed within the tumors (Figure 3A). Similarly, all sections were characterized by variable numbers of neoplastic cells with a membranous-to-cytoplasmic expression of CNPase (Figure 3B). Positive cells were mostly located in the superficial dermis, progressively reducing in number towards the bottom part of each nodule. 

IHC with primary antibodies directed against n-NF resulted in a highly variable number of disseminated structures resembling axons, with a focal, moderate to strong expression of this protein in the cytoplasm (Figure 3C). Furthermore, scattered similar structures displayed a focal cytoplasmic, weak to moderate immunopositivity in the cells for p-NF (Figure 3D). 

Differently from the control tissues, in which retina, skin melanophores, and hematopoietic precursor cells stained positive for PNL-2 and c-Kit/CD117, respectively, neoplastic cells were negative to both markers in all examined cases. Nonetheless, occasional PNL-2-positive melanophores and few to moderate numbers of c-Kit-positive infiltrating leukocytes were noted to be intermingling with neoplastic cells. Similarly, neoplastic cells were negative for all other markers investigated (i.e., calretinin, GFAP, EGR2/Krox20, and SOX2), although the corresponding primary antibodies cross-reacted with the expected target tissue(s) from the control goldfish (Supplementary Figure S1). 

Taken together, the results of the histochemical and IHC stains led to a diagnosis of tumors originating from the neural crest, consistent with PNSTs. These findings, in association with morphological features such as nuclear atypia, hypercellularity, and loss of neurofibroma architecture, led to a classification of the investigated tumors as neurofibromas resembling the entity known as “atypical neurofibroma” in human medicine.

## 4. Discussion

In this study, a group of six cohabiting goldfish bearing similar skin nodules was investigated. In all cases, the lesions were consistent with a spindle cell tumor, showing histochemical and immunohistochemical properties consistent with a neurofibroma, with morphological features (i.e., nuclear atypia, hypercellularity, loss of neurofibroma architecture) similar to the variant atypical neurofibroma described in human beings [49,50,57,58].

This diagnosis was in line with previous studies reporting peripheral nerve sheath tumors (PNSTs) to be among the most commonly occurring neoplasms in fishes in general [3,6,12,13,16,27], as well as specifically in goldfish [7,19,28,30,31,32]. 

The results of the cytological evaluation did not reflect the actual pathological condition underlying the skin nodules in four out of the six cases, being unable to correctly identify their neoplastic nature, probably due to the reduced exfoliation rate (i.e., the tendency of tissues and lesions to release cells during cytological sampling) previously associated with mesenchymal tumors [4,58].

In the current case series, routine histopathological evaluation classified the nodules as spindle cell neoplasms, which are very common tumors in teleosts [4,5]. The identification of the exact histogenesis of these tumors is a matter of debate in medical literature and a hard challenge to face in daily diagnostic routines, both in mammals [1,42,43,44,59] and fishes [3,4,5,7]. Indeed, a similar histopathological presentation in piscine neoplasms has been referred to different histogeneses, such as sarcoma not otherwise specified [24,45], fibroma/fibrosarcoma [27,34,35,36,37], leiomyoma/leiomyosarcoma [38,39,60], rhabdomyoma/rhabdomyosarcoma [42,60], PWT [40,41], myxoma [37], chromatophoroma [23,26,27,33], and PNST [12,13,19,27,28,30,31,32,45,47]. In this context, histochemical and immunohistochemical stains might be helpful to define the histogenesis of a neoplasm, despite not always being successful [5,26,27,39,52]. Specifically, the main histochemical and immunohistochemical features of spindle cell neoplasms in goldfish are revised in Table S4. 

According to previous reports, the presence of only scant, single, short collagen fibers scattered throughout the neoplasms and the diffused lack of muscular fibers with Azan trichrome allowed us to exclude fibroma/fibrosarcoma [27,28,32,36,37,45], and tumors of muscular origin (i.e., leiomyoma/leiomyosarcoma and rhabdomyoma/rhabdomyosarcoma) [38,39,60]. Unfortunately, and partially contrasting with a former study, α-SMA and desmin, which are expressed in the vast majority of mammal PWT [42], did not cross-react with the piscine tissue in this study [40]. However, the absence of whorls of neoplastic cells around blood vessels, a typical feature of PWT, excluded these tumors from the differential diagnoses [39,40,41], although this approach has been questioned [61,62]. The positivity of neoplastic cells for S100 protein was suggestive of a tumor originating from cells of the neural crest [39,63]. Additionally, the presence of variably sized hypocellular areas containing glycosaminoglycans or mucins, as confirmed by Alcian blue staining (pH 2.5), was in accordance with other studies reporting similar findings for different mammalian [1,57] and piscine neoplasms [47] originating from neural crest cells. Furthermore, the lack of PNL-2 and c-Kit expression suggested the possibility of excluding melanoma [26,64,65], the most common type of chromatophoroma in fishes [7,33], as the possible diagnosis. Still, the gross yellow to orange appearance of the nodules might be suggestive of the presence of erythrophores and/or xanthophores within the neoplasm. This hypothesis may be verified with ultrastructural investigations [33], which were not conducted due to the lack of adequate material for this specific analysis. According to the aforementioned findings and to the positivity of neoplastic cells for CNPase, which is specifically expressed by Schwann cells and oligodendrocytes, the present nodules were classified as PNSTs [1,51,66]. 

In the current study, perineurioma was excluded from the differential diagnosis list due to the lack of the typical whirling with a concentric arrangement of neoplastic cells around a central axon (i.e., “pseudo-onion bulbs”) [1,44,50], while the patchy distribution of S100 protein and CNPase expression were suggestive of a neurofibroma rather than a Schwannoma [48,51,67]. The irregular distribution pattern of PAS and Gomori’s reticulin stain-positive lamellar structures, occasionally lining individual neoplastic cells and interpreted as basement membrane fragments, was further suggestive of a neurofibroma [1,28,32,44,51]. Accordingly, the scattered positivity with Bielschowsky staining and IHC for neurofilaments was interpreted as suggestive of nerve fibers entrapped within a neurofibroma arising from neural reminiscences within the tissue, although this finding should not be considered pathognomonic [1,44,48,51]. In this context, the inconsistencies observed in this study among the different cases and between the different stains might be correlated to a very low number of nerve fibers entrapped within the neoplasms as well as to an irregular distribution of these nervous elements within the samples. Finally, the negativity for GFAP and calretinin in the investigated cases was also consistent with the diagnosis of neurofibroma, despite not being specifically suggestive of this diagnosis since a variable degree of GFAP positivity has been reported among mammal [1,46,56,67] and fish schwannomas [12,30]. Considered the aforementioned findings and according to the diagnostic criteria reported for domestic animals [48] and humans [49,50,57], the nodules examined were classified as localized, cellular to myxoid, atypical neurofibromas. 

In the current case, some features previously associated with MPNSTs (i.e., invasive growth, areas of increased cellularity and fascicular growth pattern, nuclear atypia, macronucleolosis, bi- and multinucleated cells) [28,30,44,45,46,51,57] and the immaturity of the tumoral Schwann cells (testified by the negativity for EGR2/Krox20) [68] were interpreted as histopathological findings consistent with the malignancy of the nodules. In this context, the presence of abundant extra- and intracellular glycogen granules could indicate the occurrence of metabolic reprogramming in tumor cells, which has been previously reported as a feature of malignancy [69]. Additionally, a second biopsy from the surgical scar of one animal revealed that the neoplasm had recurred 180 days after excision. Comparable findings were reported for dogs [59] and fishes [4,6], where recurrence has been associated with the infiltration of excision margins by tumor cells. On the other hand, the absence of neural crest stem cells within the tumor, as suggested by negativity to SOX2 [70] as well as the lack of other malignancy features previously associated with MPNST (i.e., necrosis, osteocartilaginous differentiation, high mitotic rate) [28,46,49,50,51,57] and, especially, the lack of fish necropsies, did not allow us to classify the present neoplasms as definitively malignant without any confirmation of metastatic behavior of the neoplasia. Additionally, the neoplastic nature of multinucleated giant cells observed in human neurofibromatosis type 1 has been questioned [71], being often consistent with reactive fibroblasts or dendritic cells rather than neoplastic cells. Therefore, the final diagnosis was neurofibroma with incipient malignant transformation, as previously suggested for canine PNSTs [46]. 

The current study describes, in detail, goldfish PNSTs resembling atypical neurofibroma in humans, providing the scientific community with a description that might be helpful to veterinary pathologists and clinicians facing similar or identical tumors. Indeed, many neurofibroma variants such as cellular neurofibroma, “atypical” or “ancient” neurofibroma, and the so-called “atypical neurofibromatous neoplasm of unknown biologic potential” (ANNUBP) have been reported among differential diagnoses for low-grade MPNSTs in human medicine [49,50,57]. Specifically, the last two variants are characterized by some features overlapping with those observed in the neoplasms described in the current study (i.e., nuclear atypia, hypercellularity, loss of neurofibroma architecture) [57,72]. In human medicine, the correlations between atypical neurofibromas and specific prognostic considerations and therapeutic options are still a matter of debate [50,57]. On the other hand, several studies in both the clinical field [72,73,74] and animal models [75] have recently reported that human atypical neurofibromas represent pre-malignant lesions that are able to progress to malignant PNSTs in a variable percentage of cases. In this context, the idea that atypical neurofibroma might represent a low-grade or a pre-malignant PNST variant rather than a frankly malignant PNST (also in goldfish) is supported by the apparent lack of correlation between death and gross evidence of tumor recurrence in the investigated animals, although a complete necropsy was not performed. Nonetheless, it should be remembered that atypical neurofibromas in humans are mainly reported in type 1 neurofibromatosis patients in the context of a syndrome that is genetically determined by several mutations of the neurofibromin 1 (NF-1) gene [57,74]. Although this syndrome has not been previously described in goldfish, it is noteworthy that experimentally induced zebrafish models carrying stable germline mutations in nf1a and nf1b, orthologs of the human NF-1 gene, developed different types of tumor, including MPNST [76]. Considered the above, the findings of the current study would require further investigations to verify the occurrence of prospective correlations between goldfish PNST resembling atypical neurofibroma in humans and specific prognostic considerations and therapeutic options in goldfish. These future studies should rely on a larger case series and investigate the prospective occurrence of mutations in NF-1 orthologs of goldfish and prognostic markers such as CDKN2A/p16 [49,50,57,73,74]. With this in mind, similar to what has been done in dogs [77], Ki-67 immunostaining was attempted in the current study; unfortunately, the antibody used did not cross-react with the expected target tissues from the control goldfish. In conclusion, further investigations focusing on the prognosis of different morphological variants of PNST in goldfish are warranted to validate the observations reported in the current study. 

In the current study, the common origin of all animals and the overlapping features among the tumors are highly suggestive of a common etiology for the observed lesions. On the basis of the considerations reported above, genetic causes can be hypothesized based on the observed predisposition of certain fish species to develop specific tumor types, such as PNSTs in goldfish in general [19,28] or neurofibromas in damselfish [13]. Unfortunately, the exclusive availability of FFPE samples in the current study did not allow an adequate in-depth workup of genetic mutations involved in PNST occurrence, known from human medicine [74], although the actual role of genetic mechanisms in fish tumorigenesis is still under debate [3,4,5,7,33,78]. Among the causes of tumors in fishes, viruses, mycobacteria, parasites, and chronic trauma and/or inflammation, chemicals, and UV radiation have been reported [3,4,5,7,10,11,20,21,22,23,79,80]. Although different virus families have been described as being able to induce neoplasms in fishes [3], only retroviruses have been described as a cause of sarcomas [5,20]. While an effective cause–effect relationship between a specific retrovirus and walleye dermal sarcoma has been reported [24], the presence of intratumoral retroviruses was demonstrated exclusively with ultrastructural investigations in Atlantic salmon swim bladder leiomyosarcoma, hooknose cutaneous fibroma/fibrosarcoma, and angelfish lip fibroma [81]. Additionally, it has been demonstrated that bicolor damselfish neurofibromatosis is associated with an undefined virus-like agent [82] rather than with a retrovirus, as previously supposed. Unfortunately, the unavailability of samples for ultrastructural investigations in the current study did not allow us to investigate the potential presence of viral particles, although this event has been described as a circumstantial finding rather than a final proof of virus-induced oncogenesis [3,4,20]. Additionally, the lack of commercially available riboprobes or antibodies directed against the aforementioned retroviruses, as well as the exclusive availability of FFPE samples, did not allow further investigations to demonstrate the presence of these agents. In the present cases, Ziehl-Neelsen staining excluded the presence of *Mycobacterium* spp., which have been previously reported as potential tumor promoters in experimental settings [10]. Similarly, the potential role of UV radiation in the induction of the neoplasms was excluded based on the localization of the fish’s pond, which was placed in an adequately illuminated spot that was not directly exposed to sunlight. Finally, the potential role of waterborne chemical agents could not be investigated due to the owner’s refusal to provide a water sample from the fish’s pond.

## 5. Conclusions

In conclusion, the current study reports the in-depth characterization of six skin nodules in a group of six cohabiting goldfish, which led to a final diagnosis of neurofibroma, resembling the entity known as “atypical neurofibroma” in humans. Indeed, similar to human medicine, the investigated tumors were characterized by their morphological features, such as nuclear atypia, hypercellularity, and loss of neurofibroma architecture, which can be suggested as key criteria to warrant further detailed classification of PNSTs in goldfish as well. Additionally, according to the observed findings, it can be concluded that a useful set of special stains to correctly identify PNSTs in goldfish might be represented by the combination of Azan trichrome staining and reticulin staining with immunohistochemistry for S100 protein and CNPase. On the other hand, future studies are warranted to evaluate the usefulness of additional immunohistochemical markers as well as to investigate the prognosis of PNSTs resembling human atypical neurofibromas in goldfish. The common origin of all animals and the overlapping features among the tumors were highly suggestive of a common etiology for the observed lesions. However, despite several investigations, such as Ziehl-Neelsen staining, an etiology for the neoplasms was not identifiable, pointing out the need for different types of sampling for piscine skin nodules, including those dedicated to ultrastructural and molecular investigations, with the latter possibly representing compelling alternatives to further characterize prospective mutations in goldfish NF-1 orthologs.

## Figures and Tables

**Table 1:** Overview of the histochemical findings in dermal atypical neurofibroma from six goldfish.

CASE ID	Azan Trichrome Stain ^a^	Alcian Blue Stain (pH 2.5)	PAS Stain ^c^	PAS-Diastase Stain	Gomori’s Reticulin Stain ^d^	Bielschowsky Stain ^f^	Ziehl-Neelsen
#1	Collagen: + ^b^ Muscular cells: −	++ (locally extensive, S)	++ (M)	−	++	−	−
#2	Collagen: − Muscular cells: −	−	+ (M)	−	+	−	−
#3	Collagen: + Muscular cells: −	+/++ (multifocal, M)	++ (S)	−	++/+++	+	−
#4	Collagen: + Muscular cells: −	+ (locally extensive, W)	++ (S)	−	+/++	+	−
#5	Collagen: + Muscular cells: −	−	+ (M)	−	++	−	−
#6	Collagen: + ^b^ Muscular cells: −	+ (focal, W)	+++ (S)	−	+++ ^e^	−	−

For alcian blue (pH 2.5) and PAS stains, the degree of intensity of the staining is reported in the parenthesis below the quantification of positivity. For Alcian blue staining (pH 2.5), the distribution pattern of positive elements is also reported in the same parenthesis. Legend: −, negative sample; +, occasional positivity; ++, moderate positivity; +++, marked positivity; M, moderate intensity; S, strong intensity; W, weak intensity. ^a^ For each case, the first line refers to the presence of blue-stained extracellular collagen fibers, while the second line refers to the positivity of neoplastic cells for magenta staining, which is consistent with muscular cells. ^b^ Multifocal to coalescing small depositions of deeply blue collagen fibers were present in the deepest portions of the nodule, at the interface to the excision margin. ^c^ All cases were characterized by diffuse, variably numerous intra- and extracellular PAS-positive granules. ^d^ All cases were characterized by the presence of scattered to disseminated, single, wrinkling, short fibrils, consistent with basement membrane fragments. ^e^ Numerous interlacing, long positive fibrils were also present. ^f^ Positivity refers to the presence of silver-impregnated fibrillar axonal elements.

**Table 2:** Overview of the immunohistochemical findings in skin nodules from six goldfish.

CASE ID	S100 Protein ^a^	CNPase ^b^	n-NF ^c^	p-NF ^d^	c-Kit/CD117	PNL-2	GFAP	EGR2/Krox20	SOX2	Calretinin ^e^
#1	+++	+++ (M–S)	++ (M–S)	−	−	−	−	−	−	−
#2	+	+ (W–M)	++ (S)	−	−	−	−	−	−	−
#3	++	++ (M–S)	−	−	−	−	−	−	−	−
#4	+++	++ (W–M)	−	+ (W)	−	−	−	−	−	−
#5	+	++ (W–M)	+++ (S)	−	−	−	−	−	−	−
#6	++	++ (W–M)	+ (S)	+ (M)	−	−	−	−	−	−

For CNPase, n-NF, and p-NF, the degree of intensity of the staining is reported in the parenthesis below the quantification of positivity. Legend: −, 0% positive cells; +, 1–25% positive cells; ++, 26–50% positive cells; +++, 51–75% positive cells; ++++, 76–100% positive cells; CNPase, 2′,3′-cyclic-nucleotide-phosphodiesterase; GFAP, glial fibrillary acid protein; M, moderate intensity; n-NF, non-phosphorylated neurofilament; p-NF, phosphorylated neurofilament; S, strong intensity; W, weak intensity. ^a^ All cases were characterized by variable numbers of neoplastic cells with strong cytoplasmic staining and patchy distribution throughout the section. ^b^ All cases were characterized by variable numbers of neoplastic cells with membranous to cytoplasmic positivity, mostly located in the superficial dermis and progressively reducing in number towards the bottom part of each nodule. ^c^ Positive samples were characterized by the presence of variably numerous axons or axonal fragments. ^d^ Positive samples were characterized by the presence of scattered, variably numerous axons or axonal fragments. ^e^ Only 4 tumors out of 6 have been tested due to lack of material in the sample block.

## Data Availability

No new data were created or analyzed in this study. Data sharing is not applicable to this article.

## Supplementary Materials

The following are available online at https://www.mdpi.com/article/10.3390/ani11092621/s1, Table S1: The host species and clonality of each tested primary antibody, as well as the corresponding antigen retrieval, serum blocking, dilution, secondary antibody, and both goldfish and canine positive controls used, are reported; Table S2: Overview on the cytological features of skin nodules from 6 goldfish; Table S3: Overview on the histological features of skin nodules from 6 goldfish; Table S4: Main histochemical and immunohistochemical features of spindle cell neoplasms in goldfish, as previously reported in the literature. Figure S1: Immunohistochemical (IHC) staining results in positive control tissues from a goldfish.
