# Supplementary material for: Peripheral Nerve Sheath Tumors Resembling Human Atypical Neurofibroma in Goldfish (Carassius auratus, Linnaeus, 1758)
Source: Animals (Basel). 2021 Sep 7;11(9):2621. doi: 10.3390/ani11092621 (PMC8467327; doi:10.3390/ani11092621)
Supplement: Supplementary file 1 [file animals-11-02621-s001.zip › animals-1309671-supplementary.pdf]

Supplementary Table S1. The host species and clonality of each tested primary antibody, as well as the corresponding antigen retrieval, serum blocking, dilution, secondary antibody, and both goldfish and canine positive controls used, are reported.

Primary antibody	Host species, clonality	Antigen retrieval	Serum blocking	Dilution	Secondary antibody (1:200)	Positive control - goldfish tissue	Positive control - canine tissue
α-SMA * (Dako – M 0831)	Mouse, monoclonal (clone 1A4), anti-human	n/a	Goat serum	1:200	GaM-b	Gastrointestinal tract	Intestine
c-Kit/CD117 (Dako – A4502)	Rabbit, polyclonal, anti-human	Citrate buffer, microwave (800 W, 20´)	Goat serum	1:100	GaR-b	Kidney – hematopoietic precursor cells	MCT
Calretinin (Synaptic systems – 214 102)	Rabbit, polyclonal, anti-mouse	Citrate buffer, microwave (800 W, 20´)	Goat serum	1:500	GaR-b	Peripheral nerve	Schwannoma
Chromogranin-A * (Dako - M 0869)	Mouse, monoclonal (clone DAK-A3), anti-human	Citrate buffer, microwave (800 W, 20´)	Goat serum	1:100	GaM-b	Pancreas – endocrine islets	Pancreas
CNPase (Chemicon International – MAB 326)	Mouse, monoclonal (clone 11-5B), anti-human	Citrate buffer, microwave (800 W, 20´)	Goat serum	1:100	GaM-b	Brain and peripheral nerve	Brain
Desmin * (Dako – M0760)	Mouse, monoclonal (clone D33), anti-human	n/a	Goat serum	1:100	GaM-b	Gastrointestinal tract	Intestine
GFAP (Dako – Z0334)	Rabbit, polyclonal, anti-bovine	n/a	Goat serum	1:1000	GaR-b	Brain	Brain

Supplementary Table S1 cont.

Primary antibody	Host species, clonality	Antigen retrieval	Serum blocking	Dilution	Secondary antibody (1:200)	Positive control - goldfish tissue	Positive control - canine tissue
Ki-67 * (Dako – M7240)	Mouse, monoclonal (clone MIB-1), anti-human	Citrate buffer, microwave (800 W, 20´)	Goat serum	1:100	GaM-b	Gastrointestinal tract	Tonsil and lymph node
EGR2/KROX-20 (LS Bio – LS-B3577)	Rabbit, polyclonal, anti-human	Citrate buffer, microwave (800 W, 20´)	Goat serum	1:500	GaR-b	Peripheral nerve	Peripheral nerve
Melan A * (Dako – M7196)	Mouse, monoclonal (clone A103), anti-human	Citrate buffer, microwave (800 W, 20´)	Goat serum	1:250	GaM-b	Eye	Melanoma
n-NF (Sternberger SMI® - SMI-311R)	Mouse, monoclonal (clone SMI 311), anti-rat	Citrate buffer, microwave (800 W, 20´)	Goat serum	1:1000	GaM-b	Brain and peripheral nerve	Brain and peripheral nerve
NSE * (Dako - M 0873)	Mouse, monoclonal (clone BBS/NC/VI-H14), anti-human	n/a	Goat serum	1:100	GaM-b	Brain and peripheral nerve	Brain and peripheral nerve
p-NF (Sternberger SMI® - SMI-312R)	Mouse, monoclonal (clone SMI 312), anti-rat	n/a	Goat serum	1:4000	GaM-b	Brain and peripheral nerve	Brain and peripheral nerve
PRX * (Sigma – HPA001868)	Rabbit, polyclonal, anti-human	Citrate buffer, microwave (800 W, 20´)	Goat serum	1:2000	GaR-b	Brain and peripheral nerve	Brain and peripheral nerve

Supplementary Table S1 cont.

Primary antibody	Host species, clonality	Antigen retrieval	Serum blocking	Dilution	Secondary antibody (1:200)	Positive control - goldfish tissue	Positive control - canine tissue
PNL-2 (Santa Cruz – SC-59306)	Mouse, monoclonal (clone PNL2), anti-Human	Citrate buffer, microwave (800 W, 20´)	Goat serum	1:500	GaM-b	Eye	Melanoma
p75^{NTR}/NGFR * (ATCC – hb-8737	Mouse, monoclonal (clone HB8737), anti-human	n/a	Goat serum	1:5	GaM-b	Brain and peripheral nerve	Brain and peripheral nerve
SOX2 (Cell Signaling Technology - #3579)	Rabbit IgG, monocolonal (clone D6D9), anti-human	Citrate buffer, microwave (800 W, 20´)	Goat serum	1:50	GaR-b	Brain and peripheral nerve	PNST
Synaptophysin * (Dako – M7315)	Mouse, monoclonal (clone DAK-SYNAP), anti-human	Citrate buffer, microwave (800 W, 20´)	Goat serum	1:500	GaM-b	Brain and peripheral nerve	Brain and peripheral nerve
S100 (Sigma – S2644)	Rabbit, polyclonal, anti-bovine	n/a	Goat serum	1:800	GaR-b	Brain and peripheral nerve	Brain and peripheral nerve
Vimentin * (Dako - M0725)	Mouse, monoclonal (clone V9), anti-porcine	n/a	Goat serum	1:100	GaM-b	Gastrointestinal tract	Intestine

Legend: α-SMA, α-smooth muscle actin; CNPase, 2´,3´-Cyclic-nucleotide-phosphodiesterase; GaM-b, goat anti-mouse biotinylated; GaR-b, goat anti-rabbit biotinylated; GFAP, glial fibrillary acidic protein; MCT, mast cell tumor; n/a, non applied or non applicable; n-NF, non-phosphorylated neurofilament; NSE, neuron-specific enolase; NGFR, nerve growth factor receptor; p-NF, phosphorylated neurofilament; PNST, peripheral nerve sheath tumor; PRX, periaxin; W, Watt.

* These antibodies did not exhibit the expected cross-reaction with the proper tissue(s) from the control goldfish and were therefore not tested on sections of the nodules.

Supplementary Table S2. Overview on the cytological features of cutaneous nodules from 6 goldfish (*Carassius auratus*).

	**Overview**				**Atypical cells**		**Cytoplasm**[f]	**Nucleus**[g]		
**CASE ID**	**Cellularity**[a]	**Extracellular matrix**[b, c]	**Extracellular red-pinkish granules**[c]	**Type of cells**[d]	**Shape (max diameter)**	**Adhesion**[e]	**Red/pink Granules**[c]	**Shape (max diameter)**	**Binucleated and multinucleated cells**[c]	**Anisocytosis and anisokaryosis**[a]
**#1**	++	+++	+++	**Atypical cells (70%)**<br>Macrophages (10%)<br>Epithelial cells (10%)<br>Melanophores (5%)<br>Lymphocytes (5%)	Spindle to polygonal (55 µm)	S/LC	++/+++	Round to oval (28 µm)	+/++	++
**#2**	++	+	+	Epithelial cells (85%)<br>Macrophages (7%)<br>**Atypical cells (5%)**<br>Lymphocytes (3%)	Spindle (45 µm)	LC	+	Oval (25 µm)	-	+
**#3**	+++	+	+	Epithelial cells (30%)<br>Macrophages (30%)<br>Lymphocytes (20%)<br>Neutrophils (10%)<br>**Atypical cells (10%)**	Spindle to polygonal to roundish (40 µm)	S	+	Round to oval (26 µm)	+ (only binucleated)	+
**#4**	+++	+	+	Epithelial cells (80%)<br>**Atypical cells (15%)**<br>Lymphocytes (3%)<br>Macrophages (2%)	Spindle (50 µm)	S/LC	+/++	Round (28 µm)	-	+

Supplementary Table S2 cont.

CASE ID	Overview			Type of cells[d]	Atypical cells		Cytoplasm[f]	Nucleus[g]		Anisocytosis and anisokaryosis [a]
	Cellularity [a]	Extracellular matrix[b, c]	Extracellular red-pinkish granules [c]		Shape (max diameter)	Adhesion [e]	Red/pink Granules [c]	Shape (max diameter)	Binucleated and multinucleated cells [c]	
**#5**	++	++	+	Epithelial cells (75%)<br>**Atypical cells (15%)**<br>Macrophages (7%)<br>Lymphocytes (3%)	Spindle to polygonal (50 µm)	S/LC	+/++	Round to oval (25 µm)	-	+
**#6**	++	+++	+++	**Atypical cells (70%)**<br>Epithelial cells (15%)<br>Macrophages (10%)<br>Lymphocytes (5%)	Spindle to polygonal o roundish (45 µm)	S/LC	+/++	Round to oval (26 µm)	++	++

[a] +, low/mild; ++, moderate; +++, high/marked

[b] Composed by a meshwork of finely fibrillar, compact, pinkish extracellular material

[c] -, none/absent; +, scant/occasional; ++, moderately abundant/moderately numerous; +++, abundant/numerous

[d] Semiquantitative evaluation

[e] S, single and sparse; LC, loosely cohesive

[f] In all cases, the cytoplasm was moderately abundant, homogeneous, and light bluish

[g] In all cases, the nucleus was centrally located, with stippled to reticular chromatin and no evident nucleoli

Supplementary Table S3. Overview on the histological features of cutaneous nodules from 6 goldfish (*Carassius auratus*).

	Growth pattern [a]			Nucleus			Atypia [c]			Additional features		Overlying epidermis	
CASE ID	Rosettes [b]	Myxoid areas [b]	Maximal cell size (µm)	Maximum diameter	Indentations [b]	Anisocytosis and anisokaryosis [d]	Nuclear atypia [b,e]	Macronucleolosis [b]	Bi-/trinucleated cells (multinucleated giant cells) [b]	Inflammation [d]	Hemorrhages [b]	Hyperplasia [d]	Erosions and/or ulcers [d]
**#1**	-	++	35 x 15	12 µm	+	++	+	-	-	-	-	-	+++
**#2**	-	-	42 x 15	16 µm	++/+++	+++	+/++	++	+/++	+	-	-	++
**#3**	+/++	+/++	30 x 18	18 µm	+	+++	++	+	+<br>(+)	++	+	++	-
**#4**	-	+	20 x 10	15 µm	+	++	++	+/++	-	+	-	+++	-
**#5**	-	-	20 x 9	12 µm	-	++	-	-	-	-	-	-	+++
**#6**	-	+	40 x 15	22 µm	+	+++	++/+++	-	-	+/+++	++/+++	+	-

[a] In all cases, the majority of neoplastic cells was arranged in streams, loosely-compact interlacing bundles, whorls, and in a storiform pattern, with occasional areas characterized by increased cellularity and fascicular pattern.

[b] -, none; +, scant/occasional; ++, moderately abundant/numerous; +++, abundant/numerous

[c] Mitotic count ranged from 0 (cases 1, 4, and 5) to 1 (cases 2, 3, and 6) per 10 high power fields.

[d] +, low/mild; ++, moderate; +++, high/marked

[e] Defined as large, hyperchromatic nuclei with smudgy chromatin.

Supplementary Table S4. Main histochemical and immunohistochemical features of spindle cell neoplasms in goldfish (*Carassius auratus*) previously reported in the literature.

**Origin**	**Histo- or cytotype**	**IHC or HC**	**Results (color in HC)**	**References**
Vessels	Haemangiopericytoma	anti-α-SMA	++	Sirri *et al*., 2016
		anti-desmin	-	
		anti-S100	+	
		anti-GFAP	-	
Connective tissue	Myxoma	Masson's trichrome-stain	NR	Shokrpoor *et al*., 2016
		Alcian blue (pH 2.5)	Myxomatous stroma (blue)	
		PAS	Stroma and neoplastic cells (magenta)	
		anti-desmin	-	
	Fibroma	Masson's trichrome-stain	NR	Constantino *et al*., 1999
			Collagenous fibers (blue)	Shokrpoor *et al*., 2016
		Alcian blue (pH 2.5)	NR	
		PAS	NR	
		anti-desmin	NR	
	Fibrosarcoma	Masson's trichrome-stain	Interlacing collagen bundles (blue)	Rezaie *et al*., 2017

Supplementary Table S4 cont

Origin	Histo- or cytotype	IHC or HC	Results (color in HC)	References (Reference number)
Pigmented cells	Chromatophoroma	anti-PNL-2	2/3 cases: +<br>1/3 cases: -	Siniard *et al*., 2019
		anti-melan A	1/3 cases: +<br>2/3 cases: -	
		anti-SOX10	3/3 cases: -	
Muscle	Leiomyoma	Masson’s trichrome-stain	Tumor cells<br>(red)<br>Small amount of collagen fibers<br>(blue)	Oryan *et al*., 2015
		anti-vimentin	++	
		anti-desmin	++	
		anti-α-SMA	++	
		anti-S100	-	

Supplementary Table S4 cont

Origin	Histo- or cytotype	IHC or HC	Results (color in HC)	References (Reference number)
Peripheral nerve sheath	Schwannoma (former neurilemoma)	anti-PCNA	+	Sirri *et al.*, 2015
		anti-GFAP	-	
		anti-S100	++	
			++	Marino *et al.*, 2007
		anti-calretinin	++	
		Orcein stain	+ (positive reticulin fibers encircling neoplastic cells)	
		Masson's trichrome-stain	Lack of collagen	
		Trichrome stains	Only occasional narrow collagen bundles	Schlumberger *et al.*, 1952
		Silver stains	Numerous reticulin fibers, rare neurites	
	Malignant Schwannoma (former malignant neurilemoma)	Silver stains	Lack of neurites	
		Reticulin stain	Lack of encircling reticulin fibers	
	Neurofibroma	Silver stains	Neurites were present	
	Pigmented nerve sheath tumors	n/a	n/a	

HC, histochemistry; IHC, immunohistochemistry; n/a, not applicable/applied; NR, not reported; -, neoplastic cells negative for the tested marker (= 0%); +, variable percentage of positive neoplastic cells (but still <100%); ++, neoplastic cells diffusely positive (= 100%)

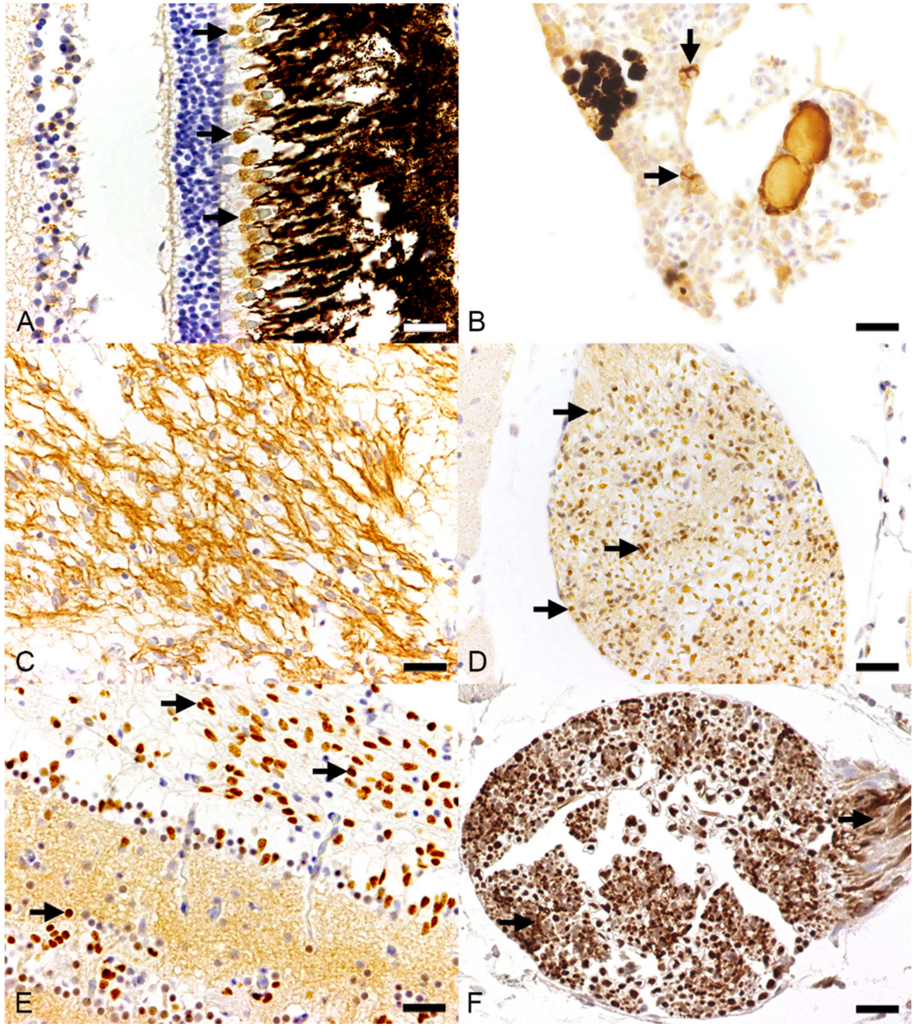

Supplementary Figure S1: Immunohistochemical (IHC) staining results in positive control tissues from a goldfish.
